# Optimizing Circulating Tumor Cells’ Capture Efficiency of Magnetic Nanogels by Transferrin Decoration

**DOI:** 10.3390/polym10020174

**Published:** 2018-02-11

**Authors:** Catalina Biglione, Julian Bergueiro, Mazdak Asadian-Birjand, Christoph Weise, Vrushali Khobragade, Govind Chate, Manoj Dongare, Jayant Khandare, Miriam C. Strumia, Marcelo Calderón

**Affiliations:** 1 LAMAP Laboratorio de Materiales Poliméricos, IPQA-CONICET, Departamento de Química, Facultad de Ciencias Químicas, Universidad Nacional de Córdoba, Haya de la Torre y Medina Allende, X5000HUA Córdoba, Argentina; catabiglione@gmail.com (C.B.); mcs@fcq.unc.edu.ar (M.C.S.); 2 Freie Universität Berlin, Institute of Chemistry and Biochemistry, Takustr. 3, 14195 Berlin, Germany; julian.bergueiro@gmail.com (J.B.); m.asadian@ymail.com (M.A.-B.); chris.weise@fu-berlin.de (C.W.); 3 Actorius Innovations and Research, B 411, GO Square, Wakad Road, 411057 Pune, India; hobragadeyvrushali@gmail.com (V.K.); drdmanoj@gmail.com (M.D.); jayant.khandare@mippune.edu.in (J.K.); 4 Surgical Oncologist, Manik Hospital and Research Center, Aurangabad 431001, India; 5 MAEER’s Maharashtra Institute of Pharmacy, Kothrud, Pune 411038, Maharashtra, India; govind@mit.com

**Keywords:** circulating tumor cells, magnetic nanogel, cell sorting, transferrin

## Abstract

Magnetic nanogels (MNGs) are designed to have all the required features for their use as highly efficient trapping materials in the challenging task of selectively capturing circulating tumor cells (CTCs) from the bloodstream. Advantageously, the discrimination of CTCs from hematological cells, which is a key factor in the capturing process, can be optimized by finely tuning the polymers used to link the targeting moiety to the MNG. We describe herein the relationship between the capturing efficiency of CTCs with overexpressed transferrin receptors and the different strategies on the polymer used as linker to decorate these MNGs with transferrin (Tf). Heterobifunctional polyethylene glycol (PEG) linkers with different molecular weights were coupled to Tf in different ratios. Optimal values over 80% CTC capture efficiency were obtained when 3 PEG linkers with a length of 8 ethylene glycol (EG) units were used, which reveals the important role of the linker in the design of a CTC-sorting system.

## 1. Introduction

The early detection of circulating tumor cells (CTCs) derived from a primary tumor due to a metastatic process is one of the hot topics in cancer theranostic [1]. A convenient approximation for detecting metastasis is to selectively trap CTCs in a simple blood test. Detection of CTCs from peripheral blood, especially when their presence is scarce, is indicative of immense prognostic value which predicts disease progression, response to treatment, and overall survival [1,2]. Different strategies have emerged in recent decades for using materials that match the requirements for capturing CTCs in blood with efficiencies ranging from 50% to 99% [3,4,5,6]. Among them, methodologies based on Velcro-like devices [7,8], biomimetic nano-platforms [9], microfluidic devices such as CTC-chips [1,10,11], DEPArray systems [12] or CTC enrichment [13,14] have been developed.

In addition, several nanomaterials such as carbon nanotubes [15], graphene-oxide nanosheets [16], nanowires and magnetic nanoparticles [17,18,19,20,21] have been investigated for the capture of CTCs. One of the most convenient approximations is the employment of magnetic nanomaterials that can selectively interact with the CTCs and sort them from a blood sample with the help of a simple magnet. These systems present several advantages such as efficient magnetic separation, the magnetic field’s harmfulness for the CTCs, easy manipulation, and their use as a magnetic resonance imaging (MRI) contrast agent [19,21]. In this sense, the CellSearch system (Menarini Silicon Biosystems Inc., San Diego, CA, USA) was the first US Food and Drug Administration (FDA) approved system for CTCs’ capture of different metastatic cancers. It consists of magnetic beads coated with antibodies against the epithelial cellular adhesion molecule (EpCAM) [22]. One relevant part of a capturing system is the targeting moiety that is able to selectively differentiate the CTCs from healthy cells. In this context, the use of transferrin (Tf) as a ligand model for the capture of CTCs has been widely studied since it is known that the concentration of the Tf receptor in many tumor phenotypic cells is considerably higher than in healthy cells [23,24,25,26,27].

Magnetic nanoparticles and targeting ligands can be also combined in a nanogel matrix that allows structural and size control in biologically pertinent nanosizes. Nanogels proved to be highly compatible in different drug-encapsulation systems between other applications. Moreover, the combination of inorganic nanoparticles with interesting physical properties can be incorporated into the nanogel network. For example, magnetic particles can be combined into a nanogel matrix forming magnetic nanogels (MNG) [28,29,30,31,32,33,34,35].

In a previous work, we demonstrated a powerful methodology for the synthesis of MNGs on the basis of an ultrasound-assisted, alkyne-azide strain-promoted cycloaddition as a crosslinking reaction [36]. Magnetic nanoparticles functionalized with bicyclononyne (MNP@BCN) and azidated thermoresponsive linear polyglycerol (tPG) were used as building blocks for synthesizing thermoresponsive MNGs. Spherically shaped MNGs were successfully obtained and decorated with Tf as a model targeting ligand. Its Tf receptor-overexpressing CTCs’ capturing ability was studied and showed trapping efficiencies up to 33%. Herein we extend our previous study, investigating the key effect of the linker used to couple the Tf ligand to the MNGs and achieve efficient CTC capture using blood samples from cancer patients. To understand and improve the system’s capturing efficiency, the interplay between the PEG linker lengths (4, 8, and 12 EG units) and the Tf-to-linker ratio (1:1, 1:3, 1:5 and 1:9) was screened.

## 2. Materials and Methods 

### 2.1. Materials

Anhydrous solvents were either purchased as ultra-dry solvent from Acros Organics^®^ (Berlin, Germany) or received from a solvent purification system. Glycidyl methyl ether (GME) and ethyl glycidyl ether (EGE) (85%, TCI Europe, Eschborn, Germany) were dried distilled, and stored over molecular sieves (5 Å). Dry toluene was obtained from MBRAUN SPS 800 solvent purification system (MBRAUN, Garching, Germany). Triethylamine (TEA, 99% Acros, Geel, Belgium), dry dimethylformamide (DMF, 99.8% Acros, Berlin, Germany), Iron (III) chloride hexahydrate (FeCl_3_·6H_2_O, 99% Grüssing, Filsum, Germany), Iron (II) chloride tetrahydrate (FeCl_2_·4H_2_O, 99% Grüssing, Berlin, Germany), ammonium hidroxide (NH_4_OH, 25% Roth, Brooklyn, NY, USA), nitric acid (HNO_3_, ≥65% Roth), picric acid solution (1.3% in water, Sigma Aldrich, Saint Louis, MO, USA), dry acetone (99% Acros, Berlin, Germany), (3-aminopropyl) triethoxysilane (APTES, 99% Sigma Aldrich), (1R, 8S, 9S)-bicyclo[6.1.0]non-4-yn-9-yl-methyl (4-nitrophenyl) carbonate (BCN-PNP), dansyl chloride (≥99% Sigma Aldrich), transferrin human (Sigma Aldrich), azido-dPEGTM12-NHS ester (Creosalus, Louisville, KY, USA), azido-dPEGTM8-NHS ester (Creosalus), azido-dPEGTM4-NHS ester (Creosalus) were used as received.

### 2.2. Methods

#### 2.2.1. Magnetic Nanoparticle (MNP) Synthesis

The synthesis was carried out following a reported precipitation method [37]. Briefly, both salts, FeCl_3_·6H_2_O (16.136 g) and FeCl_2_·4H_2_O (5.556 g) were introduced in a 250 mL round-bottom flask and 130 mL of Milli-Q water were added. The mix was stirred until all the salts dissolved. While stirring, 70 mL of NH_4_OH (3 M) were added dropwise into the solution. After 15 min, the nanoparticles were collected with a permanent magnet and the supernatant was discarded. Then, MNPs were resuspended in 20 mL of HNO_3_ (2 M) and stirred for 15 min. After rinsing with acetone 5 times, the dark compound that was obtained was stored in water. The MNPs were characterized by infra-red (IR) spectroscopy, and transmittance electron microscopy (TEM).

#### 2.2.2. APTES Modification of MNP with an Ultrasonic Horn Approach (MNP@APTES)

MNPs were modified following a previously reported methodology [36]. Briefly, 50 mg of MNPs were dissolved in 26 mL of a mixture EtOH:water (1:1). Then, 2 mL of APTES were added. The solution was sonicated five times with a horn sonicator for 2 min (70% power, Bandelin UW 2070 horn tip, Bandelin, Berlin, Germany). The mixture was then purified by washing it three times with acetone, followed by magnetic separation. Finally, the MNP@APTES were resuspended in DMF.

#### 2.2.3. Modification of MNP@APTES with BCN (MNP@BCN)

2 mg (6.34 mmol) of BCN-PNP and 200 μL of TEA were added to a 20 mL DMF solution of MNP@APTES under stirring. The reaction was carried out for 6 h. The mixture was purified by washing it three times with 30 mL DMF and magnetically separated. Then the MNP@BCN were resuspended in DMF. The MNPs were characterized by IR spectroscopy, and TEM.

#### 2.2.4. Synthesis of Linear Thermoresponsive Polyglycerol (tPG)

tPG was synthesized according to a reported methodology [38]. The reactions proceeded under argon atmosphere in a reaction flask which was dried by heating under vacuum prior to introduction of the reagents. N(Oct)_4_Br (208 mg, 0.38 mmol) was added into a flask equipped with a magnetic stirrer and the dried monomers GME (0.88 g, 10 mmol) and EGE (1.02 g, 10 mmol). The reaction mixture was cooled down to 0 °C, thus avoiding the precipitation of the ammonium salt. The polymerization was activated by the addition of *i*-Bu_3_Al solution in toluene (1 M, 1.44 mL, 1.54 mmol) and left to proceed for 16 h at room temperature. The reaction was quenched by the addition of ethanol and purified by dialysis (2 kDa molecular weight cutoff [MWCO] membrane) in toluene for 72 h. Yield: 1.67 g (83%). ^1^H nuclear magnetic resonance spectroscopy (NMR) (400 MHz, CDCl_3_): δ (ppm) = 3.89–3.94 (m, 1H, terminal OH), 3.39–3.71 (m, 409 H, polymer backbone), 3.33 (s, 105 H, –OCH_3_), 1.16 (t, 82 H, –O**CH_2_**CH_3_). FT-IR: ν (cm^−1^) = 2870, 1456, 1198, 1103, 961, 930, 872. GPC: *M*_n_ = 4746 g·mol^−1^, *M*_w_ = 5175 g·mol^−1^, *M*_z_ = 5586 g·mol^−1^, Polydispersity Index (PDI) = 1.09.

#### 2.2.5. Azidation of tPG (tPG-azide)

tPG 5 kDa (1 g, 0.2 mmol) was dried under vacuum in a 50 mL flask at 110 °C and dissolved in 7 mL of dry tetrahydrofuran (THF). Then, triethyl amine (TEA) (404 mg, 4 mmol) was added. The reaction mixture was placed on an ice bath, methanesulfonyl chloride (229 mg, 2 mmol) was added dropwise, and it was stirred at room temperature for 16 h. The ammonium salt was removed by filtration and the reaction mixture was purified by dialysis (benzoylated cellulose dialysis tubes, Sigma Aldrich, 2 kDa MWCO) against methanol for 1 day. Yellow, honey-like product was obtained. Yield: 856 mg (86%); ^1^H NMR (400 MHz, CDCl_3_): δ (ppm) = 3.39–3.71 (m, 409 H, polymer backbone), 3.33 (s, 105 H, –OCH_3_), 3.09 (m, 3H, –SO_3_–CH_3_), 1.16 (t, 82 H, –O**CH_2_**CH_3_). FT-IR: ν (cm^−1^) = 2871, 1456, 1198, 1108, 963, 930.

In a 20 mL flask, mesylated tPG (800 mg, 0.16 mmol) was dissolved in 7 mL of dry DMF. NaN_3_ (208 mg, 3.2 mmol) was added and the mixture was stirred at 60 °C for 3 days according to published procedures [38]. The salt was filtered and the reaction mixture was purified by dialysis (benzoylated cellulose dialysis tubes, Sigma Aldrich, 2 kDa MWCO) against methanol for 2 days. Yellow, honey-like product. Yield: 608 mg (77%). ^1^H NMR (400 MHz, CDCl_3_): δ (ppm) = 3.39–3.71 (m, 409 H, polymer backbone), 3.33 (s, 105 H, –OCH_3_), 1.16 (t, 82 H, –O**CH_2_**CH_3_). FT-IR: ν (cm^−1^) = 2871, 2099, 1456, 1379, 1198, 1103, 961, 882.

#### 2.2.6. Transferrin Poly(ethylene glycol) (PEG) Linker Conjugation (Tf-PEG_n_-N_3_)

To study the conjugation efficiency of the N_3_-PEGn-NHS linker to transferrin, three N_3_-PEG_n_-NHS with a different chain lengths *n* = 4, 8, and 12 were employed. For the confirmation of linker-to-transferrin conjugation efficiency, different ratios between transferrin and linker were used. The molar feed ratios of transferrin to PEG linker were 1:1, 1:2, 1:5, 1:10, and 1:20. Briefly, 25 mg of transferrin was mixed with the corresponding amount of PEG linker. 5 mL of Milli-Q water was added and the solution was stirred at room temperature for 2 h. The Tf-(PEG_n_-N_3_)_x_ conjugates were purified by dialysis (benzoylated cellulose dialysis tubes, Sigma Aldrich, 50 kDa MWCO) against water for 3 days and lyophilized. The characterization was performed by matrix-assisted laser desorption/ionization-time-of-flight mass spectrometry (MALDI-TOF-MS), circular dichroism spectroscopy (CD), and ^1^H NMR spectroscopy.

#### 2.2.7. Magnetic Nanogel Synthesis (MNG@Tf)

Ultrasound-assisted nanoprecipitation was carried out for MNG synthesis. 20 mL of Milli-Q water was heated at 50 °C in a 30 mL flask. Separately, the DMF solution containing the MNP@BCN and tPG-azide were prepared in a final volume of 1 mL. The mixture was sonicated discontinuously 6 times for 1 min using a horn sonicator (70% power, Bandelin UW 2070 horn tip sonicator). After each sonication cycle, the reaction mixture was stirred for a minute using a magnetic stirrer. After sonication 6 times, the reaction was quenched either with azido propanol or with the different conjugates, Tf-(PEG_n_-N_3_)_x_ (*n* = 4, 8, 12; *x* = 1, 3, 5, 9), to obtain Tf conjugation on nanogel surface. MNGs@Tf were purified by washing three times with acetone and magnetic separation. As a general procedure, 2 µmol of N_3_-tPG-N_3_ and 500 µL of a dispersion of MNP@BCN were used and quenched with 63 nmol of Tf-(PEG_n_-N_3_)_x_. MNGs were characterized by IR spectrscopy, nano-tracking analysis (NTA), and TEM. Tf content per nanogel was obtained via Bradford protein assay.

#### 2.2.8. Chemical Structure Characterization

^1^H NMR analysis was performed using a Bruker 400 MHz NMR spectrometer (Bruker, Billerica, MA, USA). The sample preparation, in which 8 mg of sample had been dissolved in 0.8 mL of D_2_O, was performed 24 h prior to the measurement. Fourier-transform infrared spectroscopy (FT-IR) analysis was carried out using a JASCO FT-IR 4100 LE spectrophotometer (Jasco, Oklahoma City, OK, USA) in the range of 4000–500 cm^−1^. Ultraviolet–visible (UV-Vis) absorption measurements were performed at 25 °C on a Cary 100 Bio UV-Vis spectrophotometer equipped with temperature control (Agilent, Santa Clara, CA, USA). For circular dichroism (CD) spectroscopy, samples were analyzed in 10 mM phosphate buffer (pH 7.4). CD spectra were recorded on a J-810 spectropolarimeter (Jasco, Oklahoma City, OK, USA) equipped with a temperature-controlled quartz cell of 0.1 cm path length. The recorded spectra were evaluated with the Jasco software package (Spectra Manager v3.0, Jasco, Oklahoma City, OK, USA). The spectra were averaged from three scans that were obtained by collecting data from 190 to 240 nm at 0.2 nm intervals, 2 nm bandwidth, and 1 s response time.

#### 2.2.9. Matrix-Assisted Laser Desorption Ionization-Time of Flight (MALDI-TOF) Mass Spectrometry

Conjugates were analysed by matrix-assisted laser desorption ionization-time of flight mass spectrometry (MALDI-TOF-MS) using an Ultraflex-II TOF/TOF instrument (Bruker Daltonics, Bremen, Germany) equipped with a 200 Hz solid-state Smart beam™ laser. Sinapinic acid was used as the matrix and the samples were spotted using the dried-droplet technique (sample and saturated matrix solution 1:1). The mass spectrometer was operated in the positive linear mode. MS spectra were acquired over an *m*/*z* range of 3000–100,000 Da and data was analysed using FlexAnalysis 2.4. software (Bruker, Billerica, MA, USA) provided with the instrument. 

#### 2.2.10. Dynamic Light Scattering (DLS)

Volume phase-transition temperatures (VPTT) of the samples were performed on a Malvern Nano-ZS 90 equipped with a He–Ne laser (λ = 633 nm) (Malvern, Malvern, UK) under a scattering angle of 173° at 25 °C. All the samples were maintained for stabilization at the designed temperature for 1 min before testing. Particle sizes and size distribution are given as the average of 3 measurements from the intensity distribution curves. Volume phase transition was measured at various temperatures ranging from 15 to 80 °C by DLS. 

#### 2.2.11. Nano Tracking Analysis (NTA)

NTA measurements were performed on a Malvern NanoSight NS500 (Malvern, Malvern, UK) equipped with a SCMOS camera (Malvern, Malvern, UK) and a 635 nm laser under a scattering angle of 100° at 25 °C. Video capturing was performed with a camera level, slider shutter, and slider gain set at 13, 800, and 350, respectively. For each sample, 3 videos of 30-s at 25 FPS were recorded. Analysis was carried out using the NTA 3.0 0064 software (Malvern, Malvern, UK).

#### 2.2.12. Transmission Electron Microscopy (TEM) and Scanning Electron Microscopy (SEM)

Transmission and scanning electron microscopy samples were prepared by blotting samples on carbon-coated copper grids (300 meshes, QUANTIFOIL, Grosslöbichau, Germany) and visualized by using the TEM or SEM detector on a Hitachi scanning electron microscope (SU8030, Hitachi, Tokyo, Japan) at 20–30 kV and 10 µA. 1 mg·mL^−1^ solution of each nanogel was employed.

#### 2.2.13. Cell Culture

HCT116 cells were procured from (ATCC, Manassas, VA, USA) and cultured in DMEM (Invitrogen, Carlsbad, CA, USA) and IMDM (Invitrogen) respectively, supplemented with 10% fetal bovine serum (Invitrogen), 100 units mL^−1^ penicillin, and 100 μg mL^−1^ streptomycin (Invitrogen). Blood from a healthy volunteer was obtained with protocols and guidelines. All cancer patient samples included in this study were collected at Manik Hospital and Research Center, Aurangabad, India with ethics committee approval for the study and consent forms (no number allotment). The CTC study strictly adhered to the approved protocols and the guidelines of the ethics committee.

#### 2.2.14. Isolation of Human Peripheral Blood Mononuclear Cells (hPBMC)

For isolation of human peripheral blood mononuclear cells, Ficoll-Hypaque gradient centrifugation was employed. Whole blood from healthy individual volunteers was collected into sterile heparinized vacutainer tubes. In 50 mL test tube, equal volumes of blood and phosphate-buffered saline (PBS) were mixed gently with a sterile pipette. The mixture was slowly overlaid on to 15 mL of Ficoll-Hypaque solution. It was then centrifuged at 1500 rpm for 40 min at 18 °C. The upper layer (containing plasma and platelets) was removed with a sterile pipette. The layer containing the hPBMCs was transferred into a fresh tube, washed twice with 20 mL PBS, and resuspended into RPMI complete media. Cells were counted, and the viability of the cells was determined by the trypan-blue exclusion test.

#### 2.2.15. MNG@Tf-Cell Interaction and Imaging

HCT116 cells were plated at a density of 2 × 10^5^ mL^−1^ on glass coverslips in 35 mm culture dishes for 24 h. The cells were treated with 100 μg·mL^−1^ of MNG@Tf in a time-dependent manner (5, 15, 30 min, 1, 2, 6, and 24 h). Cover slips were removed after consecutive time points and processed for confocal microscopy. Cells were fixed with 2.0% paraformaldehyde for 15 min at room temperature followed by permeabilization with 0.1% Triton X-100 in PBS for 5 min. Then, cells were washed three times in PBS and cover slips were mounted in UltraCruz mounting media with DAPI (Santa Cruz, CA, USA) and examined under an optical microscope at 63× magnification. Images were acquired simultaneously using red filter for eosin, blue filter for DAPI and green fluorescence filter to acquire anti cytokeratin (CK 18) acquisitions (to visualize MNGs). HCT116 cells were similarly treated with MNGs for 5 min, fixed, and imaged under a confocal laser microscope (LSM 510 version 2.01; Zeiss, Thornwood, NY, USA).

#### 2.2.16. Estimation of Capture Efficiency from Artificial Circulating Tumor Cells (CTC) Suspension

Artificial CTC samples were prepared by spiking hPBMCs with GFP-labeled HCT116 cells at specific ratios (1:50–1:10 5HCT116:hPBMC). 100 μg mL^−1^ MNG@Tf was added to the artificial CTC mixture, incubated for 5 min, and then subjected to magnetic field separation. The cell pellet obtained after a minute in the strong magnetic field was removed from the remaining cell suspension (containing hPBMCs and uncaptured cancer cells). Then, the images of these cells were subsequently captured and analyzed for the presence of MNG@Tf. The captured cell pellet and the residual cell suspension were imaged and the number of HCT116 cells was counted using ImageJ cell-counter plugin (open source).

#### 2.2.17. CTC Capture Efficiency in Patient Blood Samples

The breast cancer patient blood sample was treated with red blood cell lysis buffer for 15 min and centrifuged at 10,000 rpm for 10 min. The supernatant was collected; 20 µg of MNG@Tf were added, incubated for 5 min, and then subjected to magnetic separation. Cell pellets of captured CTCs were separated under magnetic field. The captured and uncaptured fractions were stained with CK 18 antibody and using the nuclear-staining probe DAPI. Captured CTCs were counted by fluorescence microscopy imaging.

## 3. Results and Discussion

### 3.1. MNP@BCN Synthesis

MNP@BCN were chosen as a building block of the MNG@Tf due to their unique superparamagnetic properties. Firstly, bare MNPs were synthesized by the co-precipitation method reported by Liu et al. [37]. Spherical particles with low polydispersity were obtained with an average size of 5 ± 2 nm measured by TEM. FT-IR spectroscopy demonstrated the presence of magnetite due to the band at 590 cm^−1^ corresponding to the Fe–O tension of the magnetite (Figure S1). In a second stage, MNPs were modified with APTES using an ultrasound-assisted method previously reported by our group [36]. We found that the size of the nanoparticles was not altered by the modification with the silane agent, maintaining an average size of 5 ± 2 nm (Figure S2a). The incorporation of the silane agent APTES into the MNP was corroborated by FT-IR spectroscopy (Figure S2b). In addition to the characteristic signals of the Fe–O of the magnetite, 3 new signals were observed around 1200 cm^−1^, corresponding to the Si–O tension of silanes. Likewise, the characteristic signals of the silane carbon chain can be seen at 2916 and 2871 cm^−1^ due to the C–H aliphatic stretch, and a band at about 3500 cm^−1^ corresponding to the amine group. Finally, MNPs@APTES were decorated with bicyclononyne (BCN) moieties through nucleophilic attack of the APTES terminal amine to BCN-paranitrophenyl carbonate (BCN-PNP) resulting in carbamate formation between MNP@APTES and BCN. The size of the MNP@BCN was not altered by this modification (Figure S3a). The reaction was confirmed by FT-IR due to the presence of the signal at 2100 cm^−1^ corresponding to the C–Csp stretch of the alkyne group in addition to the silane and nanoparticle characteristic bands (Figure S3b).

### 3.2. Thermoresponsive Linear Polyglycerol Synthesis (N_3_-tPG-N_3_)

Polyethers such as linear thermosensitive polyglycerol (tPG) are suitable candidates for the design of nanodevices due to their biocompatibility [39,40] and versatility [41,42,43]. In particular, tPGs based on glycidyl methyl ether (GME) and ethyl glycidyl ether (EGE) exhibit a thermosensitive behavior and their cloud point temperature (*T*_CP_) can be tuned between 15 and 57 °C by changing the molar ratio of GME and EGE [40,44]. Therefore, due to its outstanding properties, tPG was chosen as a building block for hybrid nanogels’ formation. We followed our previously reported protocol for the tPG synthesis [38]. The synthesis of tPG diazide (N_3_-tPG-N_3_) was performed by ring-opening polymerization of GME and EGE and subsequently followed by mesylation and diazidation of the polymer endings. The presence of the azide groups was confirmed by FT-IR due to the appearance of the signal characteristic of the azide group at 2100 cm^−1^. N_3_-tPG-N_3_ of two different molecular weights of 5 and 15 kDa was synthesized and characterized by ^1^H NMR, obtaining the characteristic polymer signals. The *M*_w_ was corroborated by GPC. The thermosensitive behavior of the different synthesized polymers was checked by turbidimetry, obtaining a *T*_CP_ of 40 °C.

### 3.3. Transferrin (Tf) PEG Linker Conjugation (Tf-PEG_n_-N_3_)

To covalently attach the Tf to the MNGs, we chose a strategy that implied the use of a linker that guaranteed enough freedom to the protein when it was attached to the MNGs to interact with the cellular receptors [45,46]. We proposed heterobifunctional PEGs of different lengths as linkers and with azide and *N*-hydroxysuccinimide (NHS) as functional groups on the polymer endings. NHS was used to conjugate the linker to the lysine residues of the Tf by amide bonding, whereas the azido groups were employed to couple by strain-promoted cycloaddition to the BCN moieties from the MNGs that were not involved in its formation.

**Figure 1:**
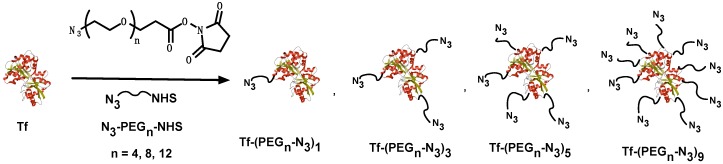
Transferrin (Tf) functionalization with PEG linkers of different sizes (4, 8, and 12 units) and in different Tf to linker ratios (1:1, 1:3, 1:5, and 1:9).

This strategy allowed us to screen several parameters on the linker like, i.e., its length, which directly affected the distance between the Tf and the MNG and therefore the Tf interaction with the protein receptor. Consequently, different azido-PEG-*N*-hydroxysuccinimide linkers (N_3_-PEG_n_-NHS) were used with different lengths of ethylene glycol chains (*n* = 4, 8, and 12) (Figure 1).

Another key factor that was analyzed by using this strategy was the number of linkers that could be used to conjugate the Tf to the MNGs. To understand its effect, 5 conjugation feed ratios’ Tf-linker were analyzed (1:1, 1:2, 1:5, 1:10, and 1:20). The conjugates were characterized by ^1^H NMR, FT-IR, MALDI-TOF-MS and CD (Figures S4–S7). Conjugation was confirmed by FT-IR spectroscopy due to the presence of the signal corresponding to the azide group at 2099 cm^−1^. The MALDI-TOF and ^1^H NMR spectra showed that the effective conjugation values for all the ratios were 1:0.5, 1:1, 1:3, 1:5, and 1:9. Therefore, a conjugation efficiency of 50% was achieved for all cases regardless of the linker’s length or the used Tf:linker ratio. To analyze whether the tertiary structure of the protein was affected by conjugation with the linker, CD measurements were performed for all conjugates. No changes on the CD signal were observed, which most likely indicated that the conjugation had no influence on Tf’s structural integrity.

### 3.4. MNG@Tf Synthesis

**Figure 2:**
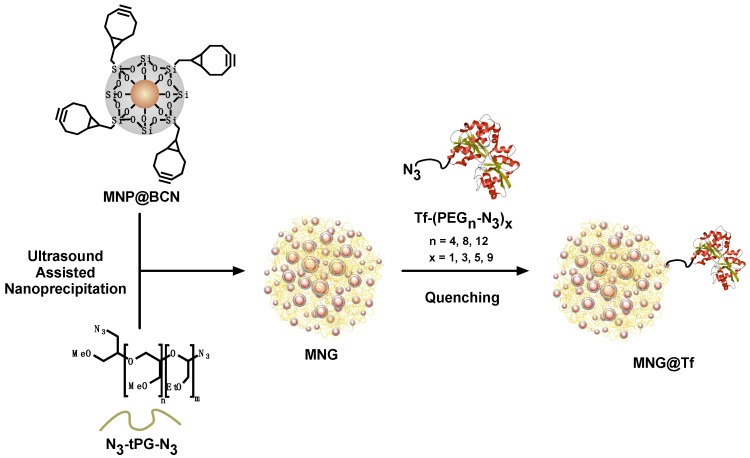
MNGs@Tf synthesis by ultrasound-assisted nanoprecipitation and its functionalization by quenching with PEG-azido-modified Tf.

For the synthesis of MNGs, we used a methodology developed in a previous work that consisted in a novel ultrasound-assisted technique based on thermonanoprecipitation [36]. This surfactant-free method allowed the synthesis of MNGs with controlled size and in short reaction times such as 15 min. The use of a tip ultrasonicator allowed control of the aggregation of the system in comparison with conventional agitation or ultrasonic bath-assisted synthetic methodologies. MNGs were synthesized through copper-free strain-promoted azide-alkyne cycloaddition between the azide groups of the polymer and the BCN groups of the nanoparticle. The remaining BCN groups were quenched with the Tf conjugate (Figure 2). Table 1 summarizes the sizes and the Tf amount of the obtained MNG@Tf with 5 and 15 KDa tPG and quenched with different linker lengths and Tf to linker functionalization ratios, along with their respective CTC capturing efficiency (Figure 2).

One of the **magnetic nanogels** (**MNG1**) was quenched with azidopropanol only and was used as a negative control for the Tf-functionalized magnetic nanogels (**MNG@Tf 1**–**MNG@Tf 13**) (Table 1). All the nanogels were water-dispersible and with hydrodynamic diameters in the range of 100–200 nm (Figure S8a–c). The amount of transferrin present in the MNGs was determined by Bradford protein essay. We found that the amount in every case was in the range between 2 and 4 μg per mg of MNG. In addition, FT-IR spectroscopy characterization indicated that the reaction was successful due to the disappearance of the signal corresponding to the azide group (Figure S8d). All MNGs presented thermosensitive behavior with a *T*_CP_ around 42 °C.

### 3.5. CTC Capture Efficiency in an Artificial CTC Suspension

**Figure 3:**
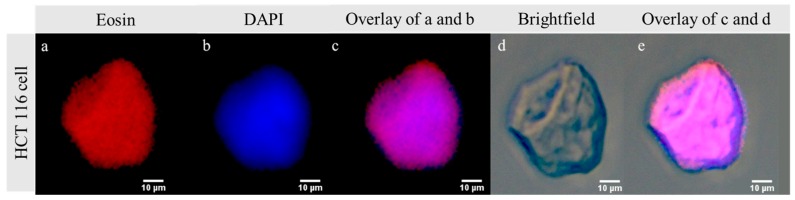
Fluorescence microscopy images of human colon cancer cell (HCT116) captured using MNG@Tf at 400× magnification. (**a**) Captured HCT 116 image under Eosin filter; (**b**) captured HCT 116 image under DAPI filter; (**c**) merged image of Eosin and DAPI filters; (**d**) bright-field image of captured HCT 116; (**e**) multichannel image of HCT 116 (**c**,**d**).

We observed the CTC capture efficiency by using MNGs mixed with human colon cancer cells (HCT 116) as an artificial suspension of CTCs and isolated after 5 min by magnetic separation. The artificial CTC suspension was prepared as reported by Banerjee et al. [25]. The HCT 116 cell line facilitate the identification and accounting CTCs without labeling with a probe or inclusion of the cytokeratin antibody (CK 18). After magnetic separation, both the captured and remaining CTCs in the suspension were imaged to estimate the number of captured and remaining CTCs in each sample. Figure 3a–e shows fluorescence microscopy images of HCT 116 cell captured using MNG@Tf. The cell-capturing efficiency was estimated based on the number of cells captured and the remnants according to the methodology reported by Zheng et al. [47]. The capture efficiencies for each MNG@Tf are detailed in Table 1.

**Figure 4:**
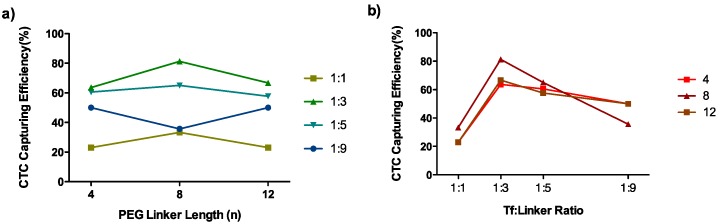
CTC (HCT 116) capturing efficiency represented against (**a**) the linker length and (**b**) the Tf to PEG linker ratio.

All designed systems could act as magnetic trap devices for circulating tumor cells. The percentages of efficiency ranged from 13% to 81% (Table 1). The linker length had a clear influence on the CTC-capturing efficiency. A length of 8 EG units showed the best results for Tf-to-linker ratios of 1:1, 1:3, and 1:5 but this tendency is inverted for a 1:9 ratio (Figure 4a). This inversion responded to the fact that an excess of linker conjugation could have a negative effect on the capturing efficiency by hindering the structure-related protein activity. This effect is clearly shown in Figure 4b where the Tf-to-linker ratio of 1:3 achieved the optimal conjugation efficiencies for all the polymer lengths. The 1:1 ratio did not guarantee effective CTC capturing, giving values of about 30%. With a ratio of 1:3, about 60% of the CTCs were captured with PEG_4_ and PEG_12_ and, even more interestingly, an 81% capturing efficiency was achieved with PEG_8_. Above a ratio of 1:3, the increase of linker per Tf decreased the capturing efficiency of the system. Thus, this behavior demonstrated that there was an optimal distance of 8 PEG units between the Tf and the MNG and an optimal Tf-to-linker ratio of 1:3, regarding the system-capture efficiency.

Since optimal parameters were found for MNGs that were composed of the building block N_3_-tPG-N_3_ with a *M*_w_ of 5 kDa (**MNG@Tf 5**), we were also interested in investigating the influence of a bigger network polymer length with respect to the capturing efficiency. Thus, **MNG@Tf 13** was synthesized according to the found parameters of 8 EG PEG linker length and a 1:3 Tf to linker ratio, but applying a tPG network polymer of higher molecular weight, 15 kDa. A tPG with a higher molecular weight is expected to generate MNGs with a bigger pore size. We hypothesize that this factor will affect the capturing ability since a bigger pore size could lead to an interaction between the Tf and the MNG pores, hindering the recognition ability [48,49]. When comparing the capturing efficiency of **MNG@TF 13** with its analogues in terms of PEG length and Tf–linker ratio (**MNG@Tf 5**), we observed a significant decrease from 81% to 22% that is in concordance with our hypothesis.

### 3.6. CTC Capture Efficiency in a Clinical Sample

**Figure 5:**
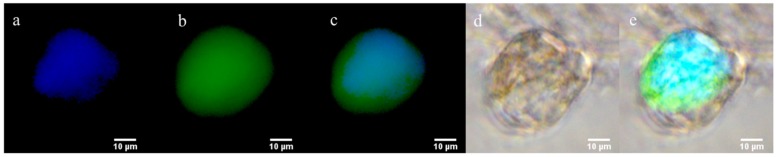
Fluorescence microscopy images of circulating tumor-cell isolation from blood sample of breast cancer patient on Tf-PEG substrate at 400× magnification under (**a**) DAPI and (**b**) green fluorescence filter due to anti cytokeratin (CK 18); (**c**) multichannel composite merged image from clinical sample (**a**,**b**); (**d**) bright-field merged image of captured cancer cell and (**e**) overlay of (**c**,**d**).

Following the influence of the thermoresponsive polymer length in capturing of cancer cells, as a proof of concept we used **MNG@Tf 5** for its ability in rapidly and efficiently capturing tumor cells from blood sample. Therefore, we evaluated a clinical sample from cancer patient’s peripheral blood. On the other hand, simultaneously we processed 1.5 mL of blood from healthy individuals as control. Tumor cells from the clinical sample were processed through immunostaining labeling and finally identified as CTCs when they stained positive for the tumor markers cytokeratin (CK 18) and DNA-interacting probe (4,6-diamidino-2-phenylindole i.e., DAPI). The images of CTCs captured from clinical samples using blood are shown in Figure 5a–e.

Captured CTCs exhibited strong CK staining and DAPI staining confirming their strong intercalation with DNA in nuclei. As anticipated, no cells were found and thus non-specific binding was not observed using Tf-decorated nanogel in any healthy blood samples. CTCs were isolated and captured due to overexpression of transferrin receptor at the surface of cancer cells. 20 µg of nanogel were able to capture 21 CTCs from 1.5 mL of patient blood, representing great CTC capturing efficiency. For the first time, the influence of polymer length along with Tf as a cancer cell-targeting moiety has been studied.

## 4. Conclusions

Using magnetic nanogels as a platform, we studied critical parameters for decorating them with a targeting moiety and optimized the capturing efficiency of circulating tumor cells. We employed a robust synthetic methodology of MNGs to investigate the effect on the capturing efficiency of different variables. The linker length and the number of linkers used to functionalize the targeting protein were very important for optimizing the system. Comparison between PEG linkers with 4, 8, and 12 EG units revealed 8 units as the optimal linker length with the best capturing efficiencies. Moreover, the spacer-to-transferrin ratio played also an important role on the system efficiency. Three linkers per transferrin gave the highest capturing values for all the polymer lengths that reached a top value of 81% for PEG_8_. Moreover, the use of a bigger thermoresponsive polymer on the construction of the MNG had a negative effect on the capturing ability of the system. The optimal linker-length polymer Tf-system was evaluated in breast-cancer patient blood, and high applicability of this system was estimated in a real scenario. Interestingly, **MNG@Tf 5** accounted for the capture of 21 CTCs from a clinical patient’s blood, implying metastatic cancer. Furthermore, CTC detection in peripheral blood indicated a prognostic value to predict disease progression, response to treatment, and overall survival.

## Figures and Tables

**Table 1:** Size, Tf amount and circulating tumor cell (CTC) capturing efficiency in an artificial CTCs suspension (HCT 116) for all MNG@Tf.

Sample	Tf-(PEG_n_-N_3_)_x_		Size (nm) ^b^	Tf Amount per MNG (µg mg^−1^) ^c^	CTC Capturing Efficiency (%)
	*n*	*x* ^a^			
**MNG1**	-	-	230 ± 90	-	13
**MNG@Tf 1**	4	1	160 ± 90	4	23
**MNG@Tf 2**	8		110 ± 90	2	33
**MNG@Tf 3**	12		120 ± 60	2	23
**MNG@Tf 4**	4	3	140 ± 70	4	64
**MNG@Tf 5**	8		110 ± 40	4	81
**MNG@Tf 6**	12		130 ± 50	6	67
**MNG@Tf 7**	4	5	150 ± 80	4	60
**MNG@Tf 8**	8		210 ± 80	4	65
**MNG@Tf 9**	12		190 ± 70	2	58
**MNG@Tf 10**	4	9	170 ± 90	2	50
**MNG@Tf 11**	8		160 ± 40	2	35
**MNG@Tf 12**	12		150 ± 90	2	50
**MNG@Tf 13 ^d^**	8	3	140 ± 40	2	22

^a^ Ratio based on mass shift detected by MALDI-TOF-MS; ^b^ Hydrodynamic diameter obtained through NTA; ^c^ Concentration obtained through Bradford protein assay; ^d^ N_3_-tPG-N_3_ with *M*_w_ = 15 kDa. For all other MNGs the polymer network N_3_-tPG-N_3_ with *M*_w_ = 5 kDa was used.

## Supplementary Materials

The following are available online at http://www.mdpi.com/2073-4360/10/2/174/s1, Figure S1: (a) TEM images and (b) FT-IR spectra of MNPs, Figure S2: (a) TEM image and (b) FT-IR spectra of MNP@APTES, Figure S3: (a) TEM image and (b) FT-IR spectra of MNP@BCN, Figure S4: ^1^H-NMR (left) and MALDI-TOF (right) spectra of Tf-PEG_4_-N_3_ conjugates in the following molar feed Tf to PEG linker ratios from up to bottom: 1:1, 1:2, 1:5, 1:10, and 1:20, Figure S5: ^1^H-NMR (left) and MALDI-TOF (right) spectra of Tf-PEG_8_-N_3_ conjugates in the following molar feed Tf to PEG linker ratios from up to bottom: 1:1, 1:2, 1:5, 1:10, and 1:20, Figure S6: ^1^H-NMR (left) and MALDI-TOF (right) spectra of Tf-PEG_12_-N_3_ conjugates in the following molar feed Tf to PEG linker ratios from up to bottom: 1:1, 1:2, 1:5, 1:10, and 1:20, Figure S7: Circular dichroism spectroscopy of Tf-PEG_x_-N_3_ conjugates, Figure S8: (a) TEM images; (b) SEM images; (c) hydrodynamic diameter distribution measured by NTA; (d) FT-IR spectra of MNG@Tf.
