# Supplementary material for: Optimizing Circulating Tumor Cells’ Capture Efficiency of Magnetic Nanogels by Transferrin Decoration
Source: Polymers (Basel). 2018 Feb 11;10(2):174. doi: 10.3390/polym10020174 (PMC6414968; doi:10.3390/polym10020174)
Supplement: Supplementary file 1 [file polymers-10-00174-s001.pdf]

polymers

MDPI

Optimizing the circulating tumor cells' capture efficiency of magnetic nanogels by transferrin decoration

Catalina Biglione,[1] Julian Bergueiro,[2] Mazdak Asadian-Birjand,[2] Christoph Weise,[2] Vrushali Khobragade,[3] Govind Chate,[4] Manoj Dongare,[3,4] Jayant Khandare,[3,4] Miriam C. Strumia,[1] and Marcelo Calderón[2,*]

Supplementary Information

<u>Bare MNPs characterization</u>

The MNPs were characterized by IR spectroscopy and TEM. Respective data is enclosed in **Figure S1**.

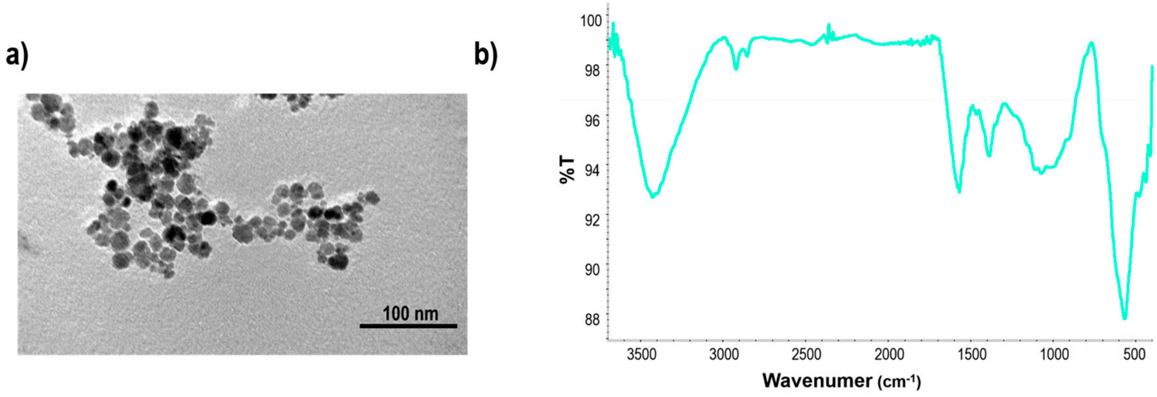

Figure S1. (a) TEM images and (b) FT-IR of MNPs.

<u>APTES modification of MNP with an ultrasonic horn approach (MNP@APTES)</u>

MNP@APTES were examined by IR spectroscopy and TEM. Respective data is included in **Figure S2**.

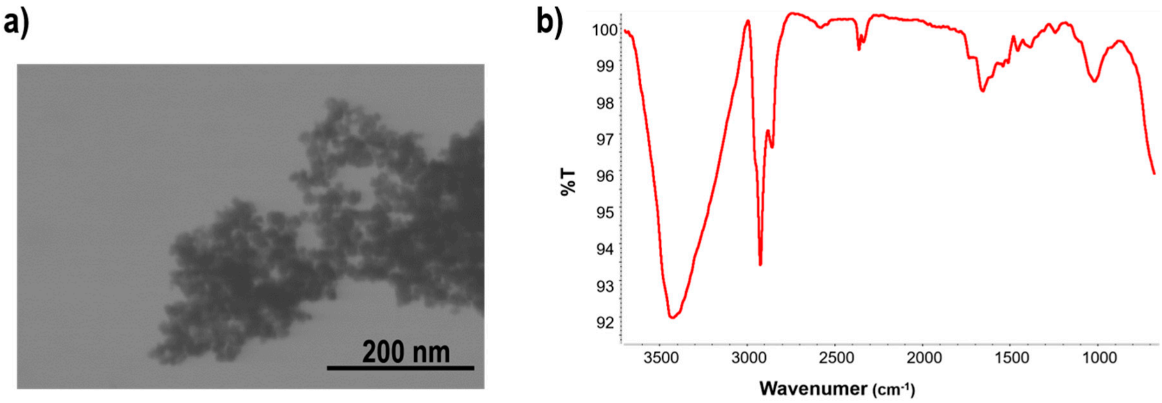

Figure S2. (a) TEM image and (b) FT-IR of MNP@APTES.

<u>MNP@BCN characterization</u>

MNPs decorated with BCN moieties were assessed by IR spectroscopy and TEM. Respective data is shown in **Figure S3**.

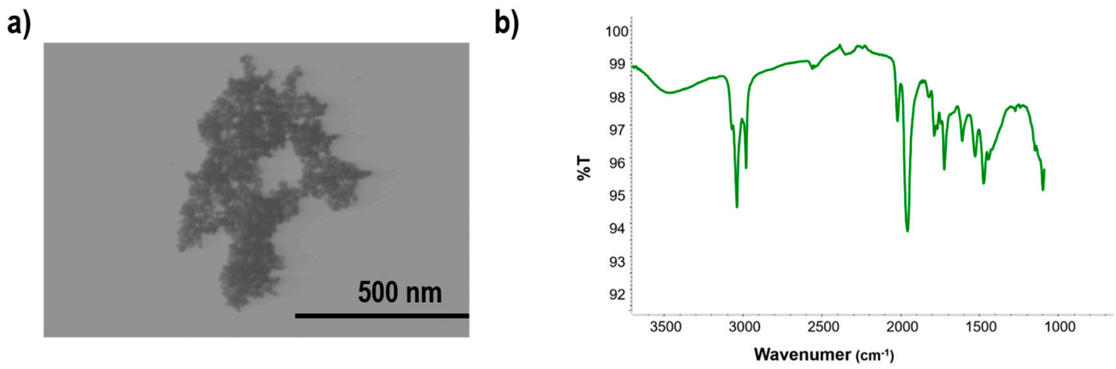

Figure S3. (a) TEM image and (b) FT-IR of MNP@BCN.

Transferrin PEG linker conjugation (Tf-PEG$_x$-N$_3$).

To study the conjugation efficiency of the N$_3$-PEG$_x$-NHS linker to transferrin, three N$_3$-PEG$_x$-NHSs were employed with different chain lengths of 4, 8, and 12. The characterization was performed by MALDI-TOF MS, CD, and ^{1}H NMR, as shown in **Figures S4 - S7**.

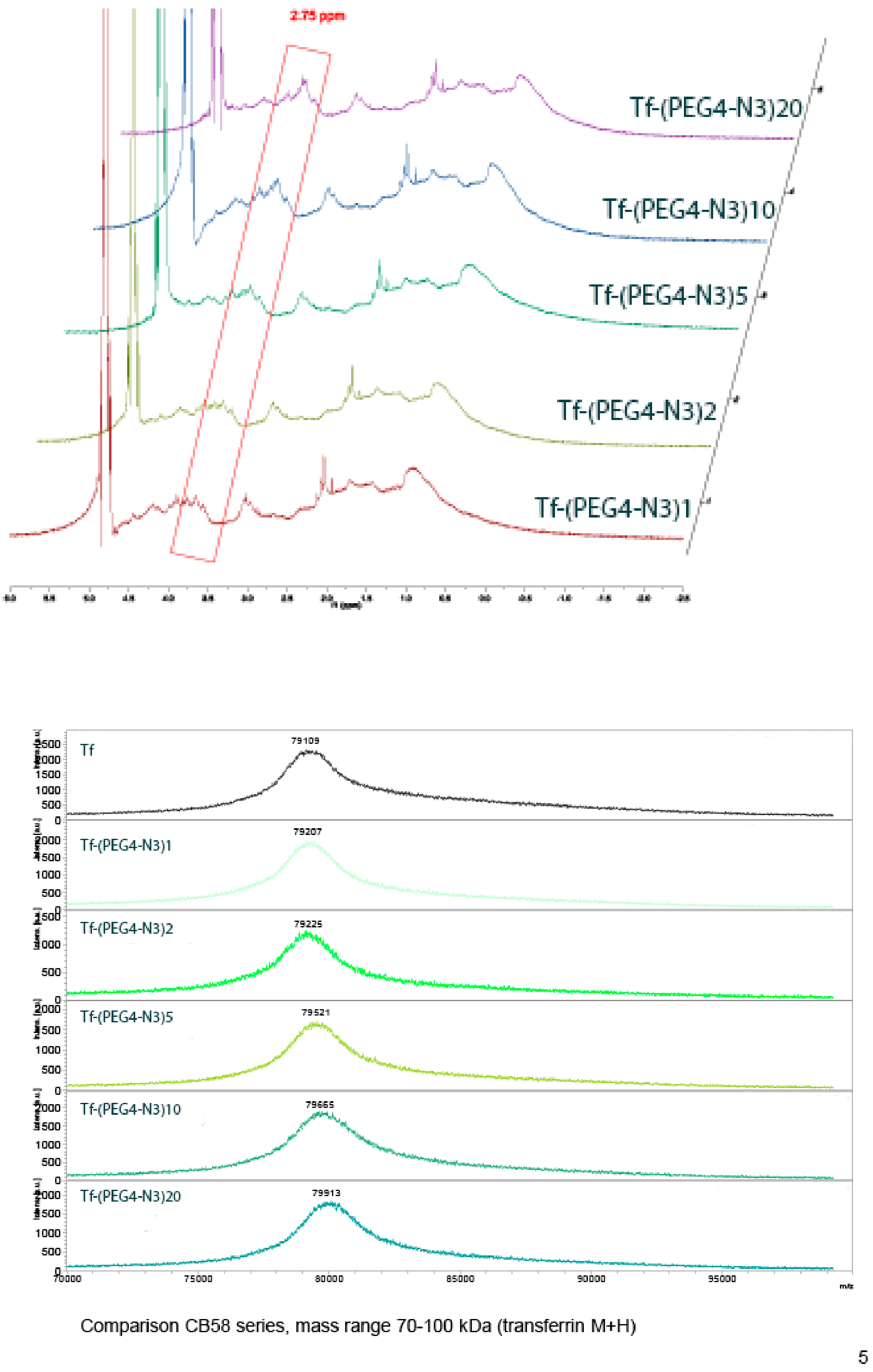

Figure S4. ^{1}H-NMR (top) and MALDI-TOF (bottom) spectra of Tf-PEG$_4$-N$_3$ conjugates in the following molar feed Tf to PEG linker ratios from up to bottom: 1:1, 1:2, 1:5, 1:10, and 1:20.

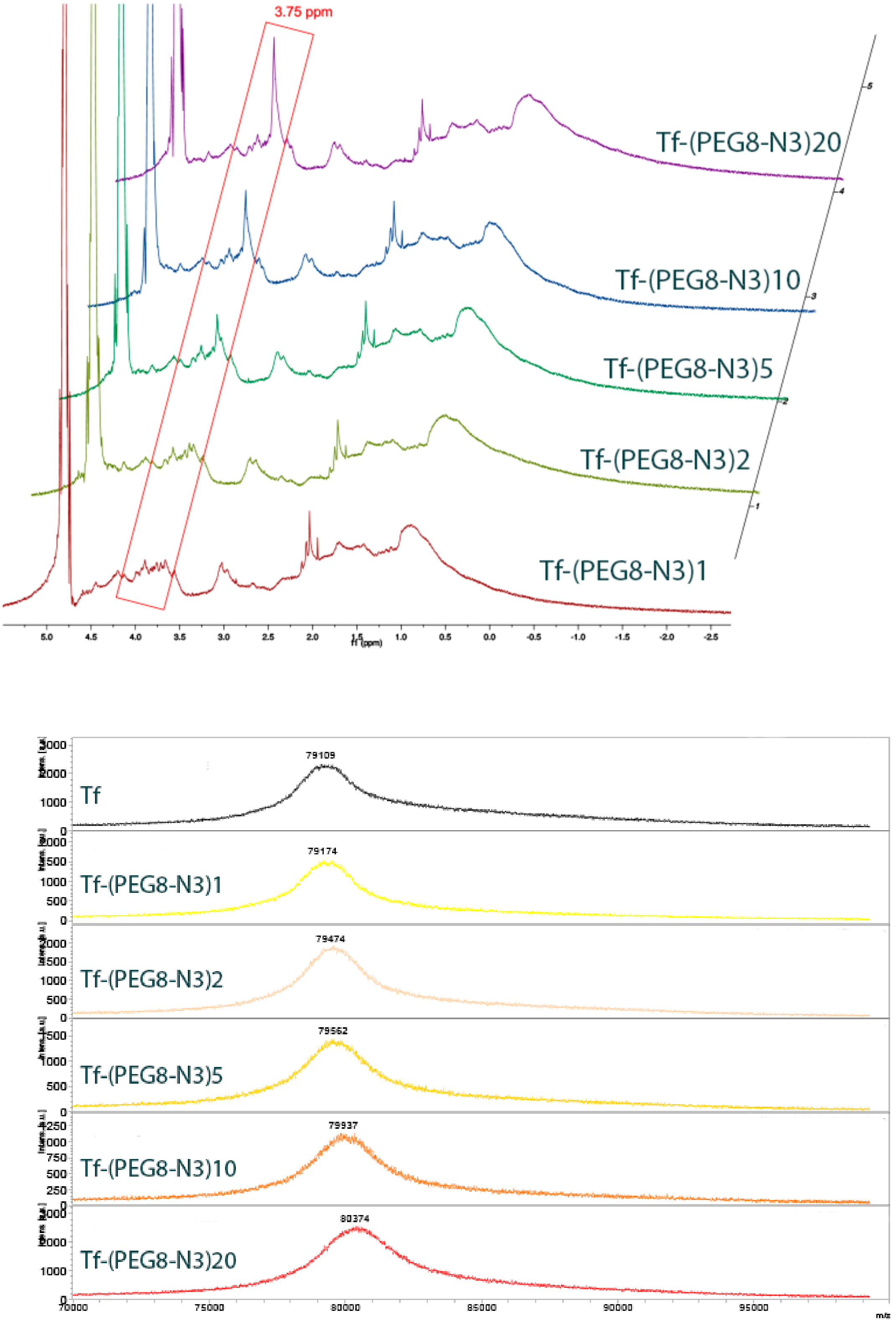

Figure S5. ^{1}H-NMR (top) and MALDI-TOF (bottom) spectra of Tf-PEG$_8$-N$_3$ conjugates in the following molar feed Tf to PEG linker ratios from up to bottom: 1:1, 1:2, 1:5, 1:10, and 1:20.

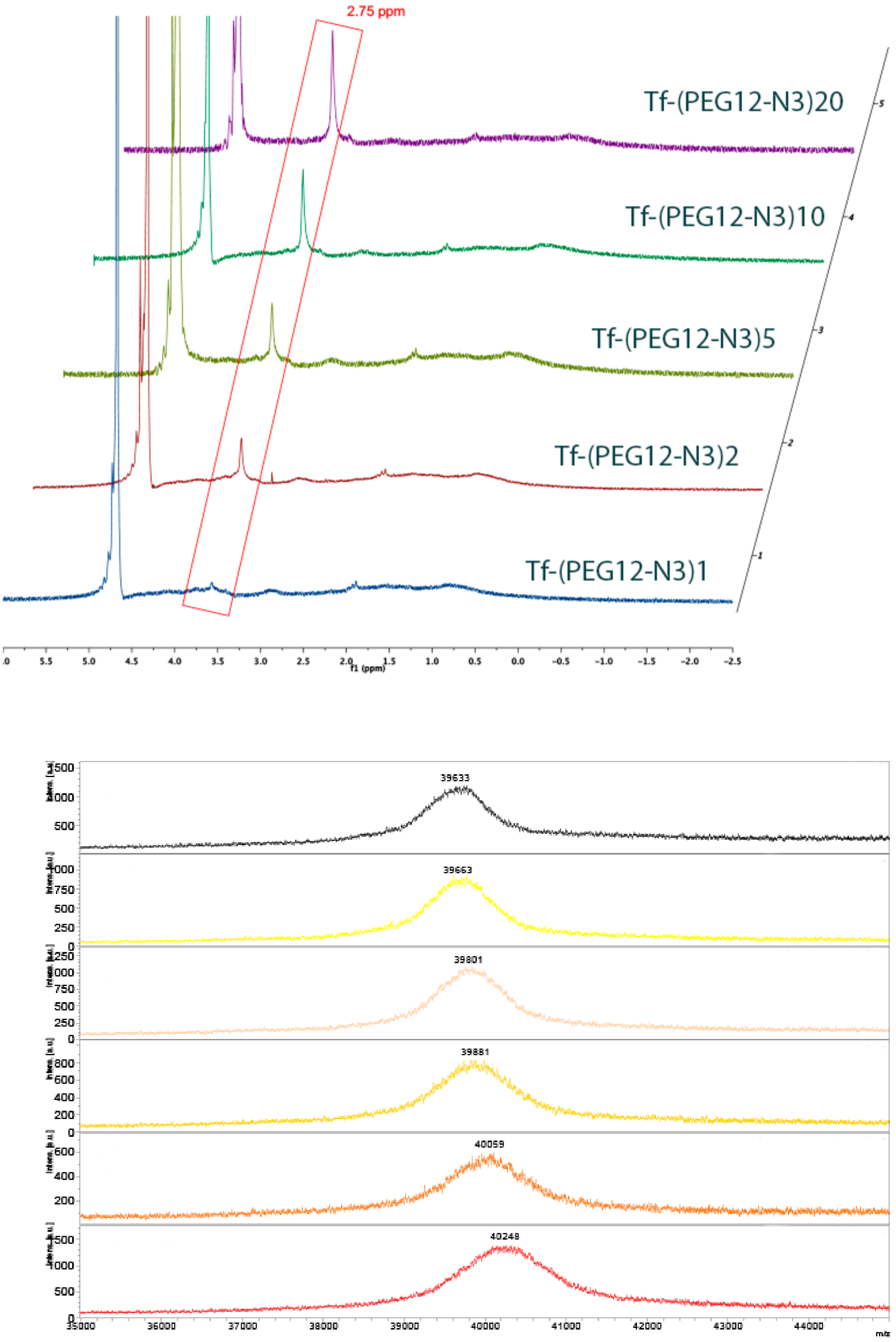

Figure S6. ^{1}H-NMR (top) and MALDI-TOF (bottom) spectra of Tf-PEG12-N3 conjugates in the following molar feed Tf to PEG linker ratios from up to bottom: 1:1, 1:2, 1:5, 1:10, and 1:20.

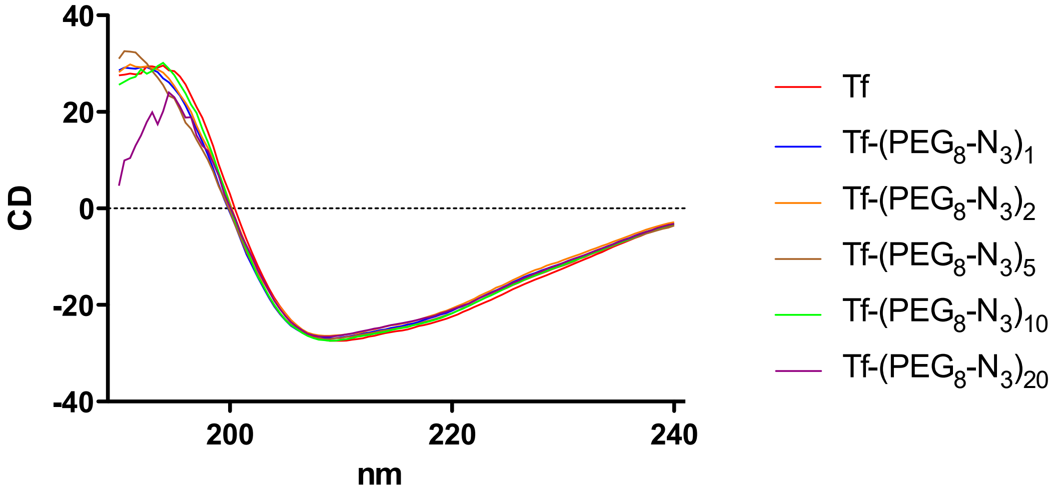

Figure S7. Circular dichroism spectroscopy of Tf-(PEG_8-N_3)$_x$ conjugates.

Nanogel characterization (MNG@Tf)

NGs were characterized by IR, NTA, SEM, and TEM. Respective data is enclosed in **Figure S8**.

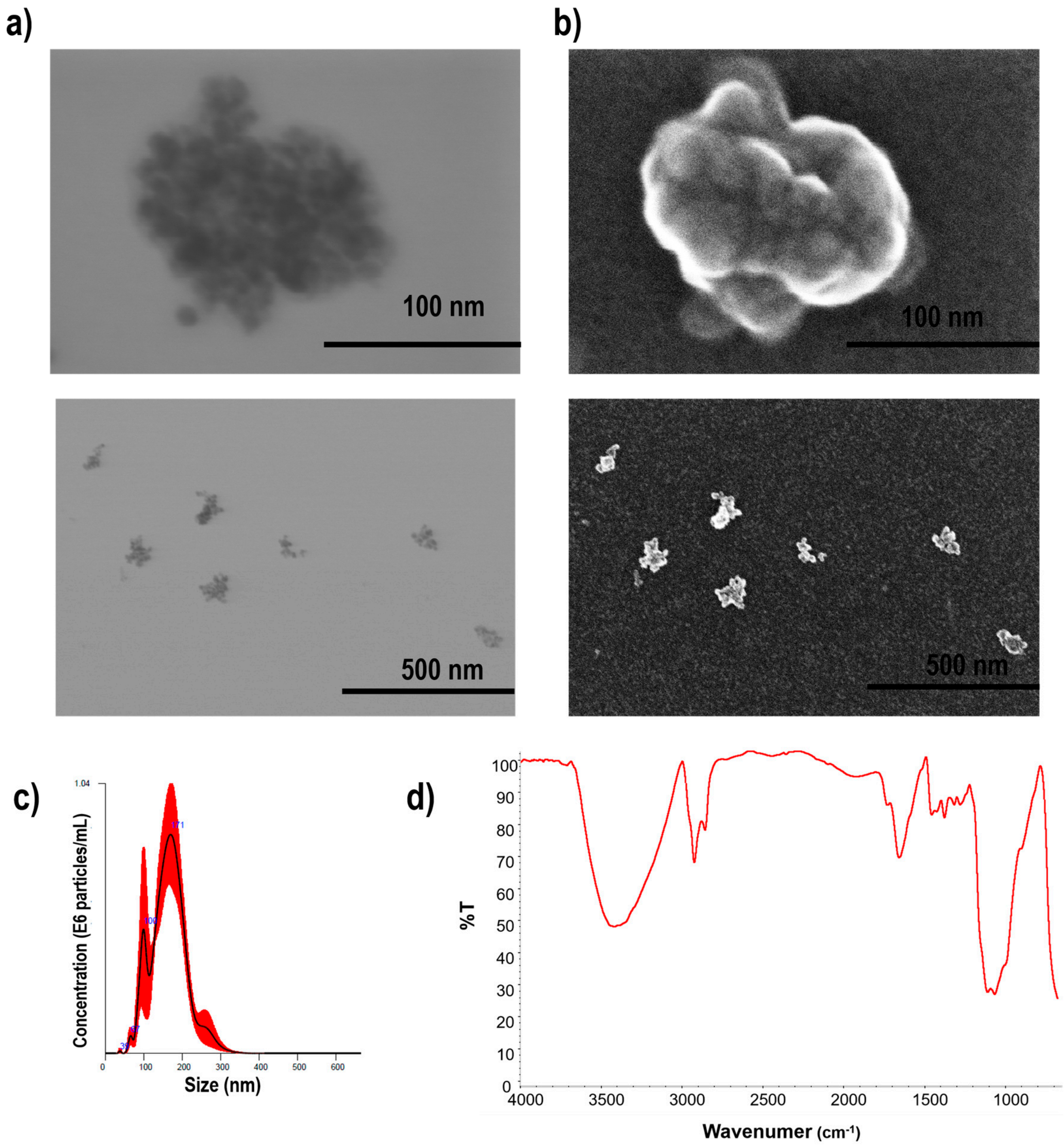

Figure S8. (a) TEM images, (b) SEM images, (c) hydrodynamic diameter distribution measured by NTA, and (d) FT-IR of MNG@Tf.
